# Safety of Boron Neutron Capture Therapy with Borofalan(^10^B) and Its Efficacy on Recurrent Head and Neck Cancer: Real-World Outcomes from Nationwide Post-Marketing Surveillance

**DOI:** 10.3390/cancers16050869

**Published:** 2024-02-21

**Authors:** Mariko Sato, Katsumi Hirose, Satoshi Takeno, Teruhito Aihara, Keiji Nihei, Yoshihiro Takai, Toshimitsu Hayashi, Kosuke Bando, Hitomi Kimura, Keisuke Tsurumi, Koji Ono

**Affiliations:** 1 Department of Radiation Oncology, Hirosaki University Graduate School of Medicine, 5 Zaifu-cho, Hirosaki 036-8562, Japan; s_mariko@hirosaki-u.ac.jp; 2 Department of Radiation Oncology, Southern Tohoku BNCT Research Center, 7-10 Yatsuyamada, Koriyama 963-8052, Japan; y-takai@hirosaki-u.ac.jp; 3 Kansai BNCT Medical Center, Osaka Medical and Pharmaceutical University, 2-7 Daigaku-machi, Takatsuki 569-8686, Japan; satoshi.takeno@ompu.ac.jp (S.T.); teruhito.aihara@ompu.ac.jp (T.A.); keiji.nihei@ompu.ac.jp (K.N.); 4 Department of Radiation Oncology, Osaka Medical and Pharmaceutical University, 2-7 Daigaku-machi, Takatsuki 569-8686, Japan; 5 Department of Otolaryngology Head and Neck Surgery, Osaka Medical and Pharmaceutical University, 2-7 Daigaku-machi, Takatsuki 569-8686, Japan; 6 Stella Pharma Corporation, ORIX Kouraibashi Building, 3-2-7 Kouraibashi, Chuo-ku, Osaka 541-0043, Japan; hayashi@stella-pharma.co.jp (T.H.); bando@stella-pharma.co.jp (K.B.); kimura@stella-pharma.co.jp (H.K.); 7 Sumitomo Heavy Industries, Ltd., 5-2 Soubirakichou, Niihama 792-0001, Japan; keisuke.tsurumi@shi-g.com; 8 BNCT Joint Clinical Institute, Osaka Medical and Pharmaceutical University, 2-7 Daigaku-machi, Takatsuki 569-8686, Japan; koji.ono@ompu.ac.jp

**Keywords:** boron neutron capture therapy (BNCT), head and neck cancer, reirradiation, post-marketing surveillance, safety, efficacy, survival

## Abstract

**Simple Summary:**

In post-irradiated recurrent head and neck cancer (R-HNC), the treatment safety margin is limited by accumulated toxicity. Despite the development of aggressive curative treatment methods, such as reirradiation with photon or particle beam therapy with or without chemotherapy, sufficient clinical outcomes have not been achieved in various prospective trials due to the limitations imposed by toxicity. Boron neutron capture therapy (BNCT), with its selective cell-by-cell dose delivery, may be an effective and safe treatment option for patients with a prior radiotherapy history, as the previous Phase II trial of BNCT in unresectable locally recurrent or locally R-HNC has shown encouraging results. In this study, we analyzed the results of a Japanese nationwide post-marketing surveillance of BNCT for R-HNC conducted under the national health insurance system and found that toxicity was well-tolerated with preferable efficacy. BNCT is suggested to be a promising treatment option in post-irradiated R-HNC.

**Abstract:**

Background: This study was conducted to evaluate the real-world safety and efficacy of boron neutron capture therapy (BNCT) with borofalan(^10^B) in Japanese patients with locally advanced or locally recurrent head and neck cancer (LA/LR-HNC). Methods: This prospective, multicenter observational study was initiated in Japan in May 2020 and enrolled all patients who received borofalan(^10^B) as directed by regulatory authorities. Patient enrollment continued until at least 150 patients were enrolled, and adverse events attributable to drugs, treatment devices, and BNCT were evaluated. The patients with LA/LR-HNC were systematically evaluated to determine efficacy. Results: The 162 patients enrolled included 144 patients with squamous cell carcinoma of the head and neck (SCCHN), 17 patients with non-SCCHN (NSCCHN), and 1 patient with glioblastoma. Treatment-related adverse events (TRAEs) were hyperamylasemia (84.0%), stomatitis (51.2%), sialoadenitis (50.6%), and alopecia (49.4%) as acute TRAEs and dysphagia (4.5%), thirst (2.6%), and skin disorder (1.9%) as more common late TRAEs. One- and two-year OS rates in patients with recurrent SCCHN were 78.8% and 60.7%, respectively. Conclusions: This post-marketing surveillance confirmed the safety and efficacy of BNCT with borofalan(^10^B) in patients with LA/LR-HNC in a real-world setting.

## 1. Introduction

Boron neutron capture therapy (BNCT) is a cell-selective particle therapy that relies on the ^10^B(n,α)^7^Li reaction. Due to differences in the intracellular uptake of ^10^B depending on tumor selectivity for the boron-carrying drug, the dose delivered to tumor cells is higher than the dose to surrounding normal cells [1,2,3]. BNCT has thus been expected as a treatment option, particularly for patients with recurrent cancer after radiotherapy or chemoradiotherapy. Neutron irradiation has been conventionally performed using nuclear reactors, but several major problems are encountered with their clinical use, such as the difficulty of installing these facilities in hospitals and the stable continuous supply due to the huge amount of time and labor required for inspection and maintenance. A small, accelerator-based epithermal neutron irradiation system was thus developed for installation in hospitals [4,5,6]. The first installation of such a commercial system in the world was at the Southern Tohoku BNCT Research Center in 2015, followed by the Kansai BNCT Medical Center in 2018 [7].

The most promising avenue for BNCT development lies in the treatment of head and neck cancer. Head and neck cancer encompasses malignant tumors originating in anatomical regions of the head and neck, including the nasal cavity, paranasal sinuses, pharynx, larynx, oral cavity, tongue, surrounding facial tissues, and some glandular tissues. These cancers predominantly stem from epithelial tissues, with squamous cell carcinoma (SCC) accounting for 90% of head and neck malignancies [8]. The treatment approach for head and neck cancer depends on factors such as cancer stage, location, and the general condition of the patient [9]. Common treatment options encompass surgery, radiation therapy, and systemic drug therapy. Cases of recurrence or advanced disease typically require multidisciplinary treatment. Currently, no universally accepted standard of care exists for recurrent disease in patients with post-irradiated head and neck cancer. Commonly employed strategies include surgical resection, reirradiation, and systemic drug therapy. However, the efficacies of these methods are constrained by the fact that they are implemented after initial treatments, reducing the therapeutic safety margin due to accumulated toxicity in the surrounding tissues, making adequate treatment challenging. Consequently, BNCT, with its potential for selective cell-by-cell dose delivery, may represent an efficient and safe treatment option [10,11].

A Phase II trial to evaluate the efficacy and safety of BNCT with an accelerator-based epithermal neutron irradiation system and borofalan(^10^B) for recurrent or locally advanced head and neck cancer, the JHN002 study, was conducted in 2016 [10]. While the response rate was 71%, especially in SCC with a complete response (CR) rate of 50% and good tumor control, no adverse events of Grade 4 or higher were encountered, indicating a high safety profile. These findings then led to PMDA approvals in March 2020, and BNCT for unresectable locally advanced or locally recurrent head and neck cancer was included for coverage by the national health insurance system in Japan in June 2020. However, due to the limited scope of the JHN002 study, which included only 21 patients, post-marketing safety and efficacy data needed to be collected.

This nationwide all-patient post-marketing surveillance (PMS) was conducted as a condition of approval for STEBORONINE^®^, the commercial name for borofalan(^10^B), and NeuCure™, a commercial neutron irradiation device (Sumitomo Heavy Industries, Tokyo, Japan) in response to requests from the Japanese regulatory authority, the Pharmaceutical and Medical Devices Agency (PMDA). Throughout the study period, STEBORONINE^®^ and NeuCure™ were used as the sole BNCT boron drug and treatment device approved in Japan, exclusively used for their intended purposes. The primary objective of this PMS was to validate the safety and efficacy of BNCT using STEBORONINE^®^ and NeuCure™ within real-world clinical settings for patients with locally advanced and locally recurrent head and neck cancer. This article presents the interim results of the study.

## 2. Materials and Methods

### 2.1. Study Design and Patients

This PMS was designed by Stella Pharma Corporation (Osaka, Japan) with the approval of the PMDA. The purpose of this PMS study was to investigate the safety of borofalan(^10^B) (STEBORONINE^®^; Stella Pharma Corporation) in BNCT and its efficacy in the treatment of patients with unresectable locally advanced or locally recurrent head and neck cancer (LA/LR-HNC). This prospective study was conducted in accordance with the Japanese Good Post-Marketing Study Practice guidelines. As the first commercial accelerator-based BNCT system, NeuCure™ was in operation at two facilities in Japan at the time of this report: Southern Tohoku BNCT Research Center; and Kansai BNCT Medical Center. Both facilities participated in this study.

In May 2020, all patients who received borofalan(^10^B) were enrolled. Patients diagnosed with unresectable locally advanced or recurrent head and neck cancer, free of distant metastases, able to maintain posture during treatment, and whose lesions are within the size of the BNCT field, were considered eligible for BNCT if the attending physicians at each institution determined that the procedure would be effective. Exclusion criteria were defined for each facility. At the direction of the PMDA, patients with tumor types other than LA/LR-HNC were also included for safety assessment. In patients who received retreatment with BNCT, registration was performed with each treatment, and collection of the first case report was completed at the time of the second registration. The safety of drugs, medical devices, and BNCT, and their efficacy against LA/LR-HNC were monitored for three years, and case report forms (CRFs) were required to be recorded within seven days and at six months, one year, two years, and three years after BNCT. The initial indication from the PMDA was to conduct a post-marketing use-results survey covering all patients treated with the product until a certain number of cases had been accumulated, with a rough goal of enrolling 150 cases over a two-year period. The study was conducted under contract with each participating site. The survey using CRFs was started at the BNCT facility where treatment had been performed. If observation at the BNCT facility ended after six months and a patient was transferred to another hospital, the new hospital was requested to participate in this PMS, and the survey was continued at the new hospital. If follow-up was interrupted due to death or transfer to a different hospital with insufficient cooperation, observation was terminated at that point. Since the first enrollment reached the planned 150 cases, case enrollment alone was continued without CRF survey after February 2022, in accordance with instructions from the PMDA.

### 2.2. Data Collection

Before conducting BNCT, the following information was recorded on the CRF: patient demographics; primary tumor site; site of recurrence; carotid artery involvement; TNM staging; Eastern Cooperative Oncology Group Performance Status (ECOG-PS); medical history; allergies; and comprehensive treatment history including surgery, radiation therapy, and chemotherapy at the initial presentation. Concomitant medications were also diligently documented in the CRF.

The CRF, to be completed within seven days, included the treatment date, total STEBORONINE^®^ dosage, measurement of boron concentration in whole blood both just before the start of neutron irradiation and just after the end of irradiation, documentation of neutron irradiation details (including start and end times), records of any interruptions to irradiation (including durations and reasons for such interruptions), and the planned versus actual amounts of accelerator irradiation charge. In addition, the prescription dose corresponding to the maximum dose to the mucosa in the treatment of head and neck cancer, and maximum, minimum, and average dose for the tumor were all recorded as part of the scheduled irradiation doses. Furthermore, the presence or absence of medical equipment malfunctions, the names of malfunctioning equipment, the serial number of equipment, the name of the malfunction, and the circumstances surrounding occurrences were meticulously logged.

The adverse event-reporting page accompanying all case reports contained the following information: date of adverse event onset; terminology used to describe the adverse event; grading according to the National Cancer Institute Common Terminology Criteria for Adverse Events (NCI-CTCAE), version 5.0; outcome details; date of outcome confirmation; potential causative factors for the adverse event, aside from borofalan(^10^B), NeuCure™, or BNCT; medication administered for symptoms; clinical management; and relevant laboratory values.

In the case reports after six months, the date of efficacy determination and the results of anti-tumor efficacy determination for the target lesion according to Response Evaluation Criteria in Solid Tumors (RECIST), version 1.1, were recorded in relation to clinical efficacy and survival, date of confirmed survival or death, cause of death, causal relationship between death and drugs, medical devices, and BNCT, and pre- and post-death status were recorded in relation to patient prognosis. In cases of ongoing investigation at the transferred medical facility, the name of the medical institution, department name, and the name of the responsible physician were also thoroughly examined. Patients who had been transferred to a different hospital continued to be monitored at the new facility. All PMS requests to the transferring hospitals and data collection were performed by Stellar Pharma Corporation personnel.

### 2.3. Treatments

Treatment planning was performed in RayStation (RaySearch Laboratories, Stockholm, Sweden), and the dose calculation was performed using the NeuCure™ Dose Engine (Sumitomo Heavy Industries). The parameters used in dose calculations were based on the values used in the JHN002 study that led to approval, and each facility finally adopted its preferred values [10]. Specifically, datasets of the tissue composition defined by the International Commission on Radiation Units and Measurements Report 46 were used for calculating transmutation reactions with neutrons [12]. Relative biological effectiveness factors were 1 for gamma rays, 2.4 for fast neutrons, and 2.9 for thermal neutrons. Compound biological effectiveness factors for boron derived from borofalan(^10^B) were 4.9 for mucosa, 2.5 for skin, and 1.34 for brain tissues. For tumors, a value of 3.8 or 4.0 (referring to JHN002) was selected depending on the background of each institution. Similarly, the tumor-to-normal tissue ratio of ^10^B concentration was defined constantly as 3.5 (referring to JHN002) or the empirical value of the facility.

All patients received their first intravenous administration of borofalan(^10^B) at 200 mg/kg/h for 2 h, followed by the start of the second dose of borofalan(^10^B) at 100 mg/kg/h and simultaneous neutron irradiation. Whole-blood sampling was performed 2 h after starting the first administration of borofalan(^10^B), and the concentration of ^10^B was measured with inductively coupled plasma atomic emission spectroscopy. Neutron irradiation time was determined by the whole-blood ^10^B concentration, and prescription doses were determined for each facility. Neutrons were irradiated using NeuCure™. Continuous infusion of borofalan(^10^B) was stopped at the same time irradiation was stopped, within a maximum of 1 h after the start of irradiation.

### 2.4. Assessments

CRFs were filled out five times: within seven days of BNCT; and at six months, one year, two years, and three years after BNCT. The primary outcome measure was the incidence of adverse effects. AEs were assessed using NCI-CTCAE version 5.0 and were coded by preferred terms using the Medical Dictionary for Regulatory Activities (MedDRA)/J version 25.1 by Stella Pharma Corporation personnel. AEs were classified as acute AEs if they occurred within 90 days of the date of BNCT and as late AEs if they occurred from 91 days onwards, and were regarded as late toxicities by the attending physician.

Secondary efficacy endpoints included tumor response and overall survival (OS). Tumor response was based on RECIST version 1.1 using consistent imaging with CT or MRI. The best overall response of lesions treated with BNCT was recorded at any point within six months after BNCT. The objective response rate (ORR) was evaluated as the proportion of patients showing CR or partial response (PR). Tumor response in patients receiving post-treatment intervention was also included in the analysis.

### 2.5. Statistical Analysis

Descriptive statistics were used to summarize patient characteristics and treatment parameters. The distribution of tumor response was compared using two-sided Mann–Whitney U tests. For the calculation of ORR and the corresponding 95% confidence intervals (CIs), the Clopper–Pearson method was used. The Kaplan–Meier method was used to estimate median OS. Tumor response in patients receiving post-treatment intervention was also included in the analysis.

## 3. Results

### 3.1. Patient Disposition and Baseline Characteristics

**Figure 1:**
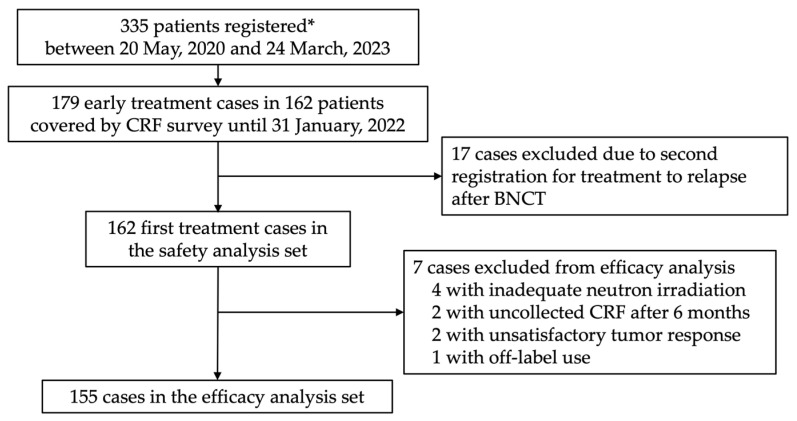
Patient disposition. * Including a glioblastoma patient. Abbreviations: CRF, case report form; BNCT, boron neutron capture therapy.

Between 20 May 2020 and 24 March 2023, 364 cases in 334 patients with unresectable LA/LR-HNC and one patient with glioblastoma were registered from the two facilities. Of these, a total of 179 cases from the initial 162 patients treated until 31 January 2022, were included in the survey using CRFs. At least one CRF was collected from all these patients (Figure 1). Collection rates for CRFs are shown in Table 1. These 162 cases were used in the safety analysis. One case received administration of borofalan(^10^B) but did not undergo neutron irradiation. Six patients were excluded from the safety analysis set, and a total of 155 cases were finally included in the efficacy analysis. Reasons for exclusion were as follows: one case was used off-label for glioblastoma; two cases showed no CRF recovery after six months of BNCT; two cases displayed unknown or unevaluated tumor response; four cases received inadequate neutron irradiation with >2% deviation from dose planning; and two cases had multiple reasons.

The baseline characteristics of patients who were eligible for analysis are shown in Table 2. Patients enrolled twice had characteristics as of the first enrollment listed. The underlying histology was SCC in 144 patients and non-SCC (NSCC) in 17 cases. No patients showed PS ≥ 3, with the potential exception of one patient with unknown PS. Among all patients, 151 patients had a history of at least one course of radiotherapy or chemoradiotherapy to the head and neck. Total dose in this table represents the sum of multiple radiation doses to the head and neck region, with a median cumulative dose of 70 Gy (range, 24–130 Gy).

### 3.2. Status of BNCT Implementation

Treatment-related parameters are summarized in Table 3. The ^10^B concentration in whole-blood at 2 h after the start of borofalan(^10^B) administration exceeded 20 ppm in 161 patients, excluding one patient for whom treatment was cancelled during the borofalan(^10^B) administration phase, with a median value of 31.3 ppm (range, 20.9–46.5 ppm).

Neutron irradiation was continued until the prescribed mucosal dose was achieved. The prescribed mucosal dose was based on 12 Gy-Eq (referring to JHN002) and was adjusted at the discretion of the attending physician. The median value was 15 Gy-Eq (range, 5–20 Gy-Eq). Maximum, minimum, and average tumor doses at planning were 47.0 Gy-Eq (interquartile range [IQR], 38.9–56.6 Gy-Eq), 26.9 Gy-Eq (IQR, 22.5–32.3 Gy-Eq), and 38.8 Gy-Eq (IQR, 32.3–45.5 Gy-Eq), respectively. Consequently, the accelerator proton charge used for neutron irradiation was 2.43 C (IQR, 2.03–2.95 C) and included five cases with unplanned interruption in the neutron irradiation phase (3.1%).

### 3.3. Safety

One patient who received only drug administration and no neutron irradiation was included in the safety assessment at the direction of the PMDA. Treatment-related AEs (TRAEs) related to BNCT were reported in 162 cases (Table 4). The most frequent TRAEs were salivary gland hyperamylasemia in 136 patients (84.0%). Except for hyperamylasemia, Grade 3 and above were observed in 58 cases (35.8%), Grade 4 in 12 cases (7.4%), and Grade 5 in one case (0.6%). One patient with hypophagia did not recover oral intake after BNCT, showed local recurrence after 201 days, and died from cancer after 242 days, but hypophagia was reported as Grade 5 because the symptom had an impact on the cause of death. With regard to acute TRAEs, 34 adverse events were reported as attributable to borofalan(^10^B), including 32 cases of crystalluria and two cases of hematuria. Stomatitis was present in 83 patients (51.2%), with symptoms classified as Grade 3 in 17 patients. Alopecia was seen in 80 patients (49.4%). Two patients developed tracheal stenosis or airway obstruction in the acute phase. The most common late TRAEs were dysphagia in seven patients (4.5%), thirst in four (2.6%), and skin disorder in three (1.9%) (Table 5). Grade 3 AEs included dysphagia in three patients (1.9%), and skin disorder, hyperamylasemia, soft tissue infection, and papillary optic nerve edema in two patients (1.3%) each. The only Grade 4 event was development of pneumonia due to aspiration in one patient (0.6%).

### 3.4. Efficacy on LA/LR-HNC

For efficacy, 406 CRFs (76.2%) at six months and beyond were collected from 155 patients with head and neck cancer eligible for analysis. Of these, the overall CR rate was 46.1% in 154 HNC patients, excluding one SCC patient with insufficient data on tumor response at six months (Table 6). For the 137 SCCHNs, the ORR was 72.3%, comprising CR in 46.0% and PR in 26.3%. For the 17 NSCCHNs, CR was seen in 47.1% and PR in 17.7%, providing an ORR of 64.7%. Patients with lymph node metastases tended to show lower CR and response rates, although no significant differences were observed (*p* = 0.2286, Table 7).

**Figure 2:**
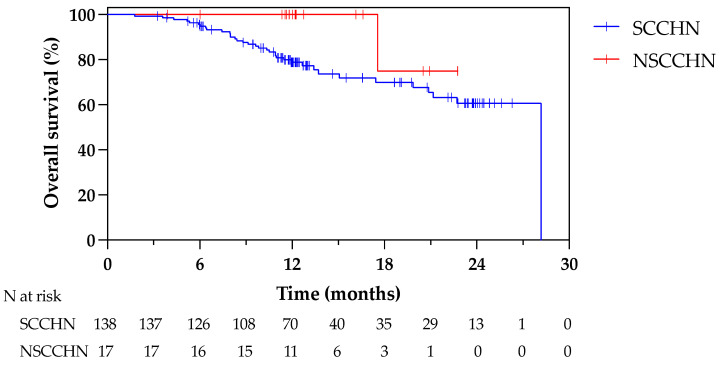
Survival curves in patients with SCCHN and NSCCHN. Abbreviations: LR, locoregionally recurrent; SCCHN, squamous cell carcinoma of the head and neck; NSCCHN, non-squamous cell carcinoma of the head and neck.

Figure 2 shows Kaplan–Meier curves for OS in patients with recurrent SCC and NSCC. For the 138 patients with SCCHN, 1- and 2-year OS rates were 78.8% and 60.7%, respectively. For the 17 patients with NSCCHN, the 1-year OS was 100%.

## 4. Discussion

Borofalan(^10^B) represents a pioneering boron-based drug that has advanced to clinical development for BNCT. It is the sole drug approved in Japan for the management of LA/LR-HNC. The JHN002 Phase II trial has confirmed the efficacy and safety profile of this drug. However, clinical evaluations of safety and efficacy will not reflect reality unless performed in situations with diverse patient characteristics, such as comorbidities, general condition, and patterns of tumor progression. Unfortunately, the population of patients with locally recurrent head and neck cancer in the JHN002 trial was relatively small and did not allow for adequate clinical evaluation of treatment. The present PMS study undertook the crucial task of assessing the safety and efficacy of borofalan(^10^B) as a therapeutic intervention for recurrent head and neck cancer within a real-world context, involving 162 patients. This comprehensive investigation encompasses a patient cohort with a variety of background characteristics, offering a more realistic representation of the general patient population. As pre-treatment, 93.2% of patients received radiation therapy and 79.0% received systemic drug therapy. These statistics reflect a substantial portion of patients who asked for BNCT as a treatment option following refractory interventions. Furthermore, 88.9% of patients presented with SCC, aligning the patient background profile with a more realistic distribution of histological types. Importantly, this study serves as a pioneering endeavor by establishing the safety of BNCT as a reirradiation strategy for patients with recurrent head and neck cancer with prior irradiation history, using an extensive real-world dataset.

The drugs used in BNCT are unique in that they contain ^10^B, and the drug itself does not exhibit any pharmacological effects. Instead, biological activity only occurs upon exposure to neutrons, initiating nuclear reactions with ^10^B. Consequently, side effects directly attributable to borofalan(^10^B) are exceedingly rare and mainly associated with factors such as crystalluria and diarrhea. The safety data included one case of glioblastoma, but otherwise consisted entirely of patients with head and neck cancer, strongly reflecting the safety profile at head and neck irradiation sites. Notably, Grade 3 or higher adverse events (e.g., stomatitis, pharyngitis, dysphagia) with reirradiation of the head and neck were revealed more clearly than in the JHN002 study. Tissue defects in the skin and mucosa that occur as a result of tumor necrosis led to Grade 3 deep tissue infections and cellulitis, which were relatively frequent. The risk of carotid artery rupture due to the spread of infection from soft tissues to the carotid artery has been reported [13]. No carotid artery rupture was encountered in this study, suggesting that appropriate decisions on indications based on patient selection criteria were achieved at each BNCT facility.

The two BNCT sites participating in this study used different values of CBE, which were determined based on the surviving fraction in rat gliosarcoma for in vivo–in vitro study, and values of 4.0 or 3.8 values were derived using 10% or 1% cell viability as endpoints. While past reports have described clinical studies in which a value of 3.8 was used, a value of 4.0 was used in JHN002 clinical trials for regulatory approval. Hence, a value of 4.0 was used in centers where insurance coverage was based on the conditions of approval, and a value of 3.8 was used in one center that had previously preferred a more frequent value. The potential difference in reported tumor doses between these centers should be taken into account and still be used as reference values for the future. The variability in BNCT in terms of boron accumulation in patient tumor tissue and responsiveness, as reflected in the CBE of individual tumors, has not yet been clarified. Hence, it is difficult to make dose prescriptions based on tumor doses according to patient and tumor characteristics. Therefore, treatment was implemented to eliminate the effects of these variations by applying a dose prescription based on the upper limit of the tolerated dose of the mucosa. Further investigation of correlations and discrepancies between the derived tumor doses and clinical response may shed some light on case-specific differences based on patient and tumor characteristics.

With recurrent head and neck cancer following standard treatment, treatment safety margins are limited due to accumulated toxicity in the treatment area. Consequently, treatment of recurrent head and neck cancer remains a challenging task. Despite numerous prospective trials conducted to date, outcomes have remained limited. Results from prospective trials on reirradiation ± chemotherapy without surgery have reported a 1-year OS of around 50% at most [14,15,16,17,18,19,20,21]. A post hoc analysis of KEYNOTE-040 failed to demonstrate the superiority of pembrolizumab over standard therapy for locoregionally recurrent SCC [22]. A prospective trial of photoimmunotherapy, which is similarly accessible under the national health insurance system in Japan as BNCT, reported a 1-year OS rate of 49.8% [23]. Conversely, retrospective analyses of particle radiotherapy for reirradiation have often reported favorable 1-year OS rates of 64–84% [24,25,26,27,28]. This study demonstrated a CR and ORR for recurrent head and neck squamous cell carcinoma using BNCT of 44.7% and 70.9%, respectively, representing favorable results when compared to previous prospective and retrospective trials and consistent with the outcomes from the limited cases registered in the JHN002 trial. This suggests that BNCT may be an effective treatment modality for recurrent head and neck SCC. In Japan, the national health insurance system has made head and neck cancer patients accessible to BNCT at an early stage of treatment at the time of recurrence, which may have contributed to this favorable outcome.

Another notable feature of this report was the presentation of a substantial amount of clinical data on head and neck NSCC. While long-term tumor control data are crucial for NSCC, the utility of this treatment could not be determined at this stage due to the relatively short observation period. For both SCC and NSCC, the observation period was too short to evaluate OS beyond two years. We therefore eagerly await the final analysis results for this ongoing investigation.

## 5. Conclusions

This nationwide PMS suggests that borofalan(^10^B) and BNCT are safe. In clinical practice, the potential efficacy of BNCT for recurrent head and neck cancer was suggested, even in patients with various characteristics, such as complications, general condition, and diverse tumor progression. The results are, to some extent, consistent with previous Phase II trials. Despite the short observation period, the PMS results, from a study of all patients, provide real-world observations of BNCT treatment in Japan.

## Figures and Tables

**Table 1:** Collection rate of survey sheets.

Period	CRF Issued, *n*	CRF Collected and Fixed, *n*	Collection Rate (%)
**Within 7 days**	179	179	100
**6 months**	198	177	99.4
**1 year**	156	142	100
**2 years**	128	76	64.4
**3 years**	51	11	22.9

Abbreviation: CRF, case report form.

**Table 2:** Baseline characteristics.

Characteristic	All (*n* = 162)	SCCHN (*n* = 144)	NSCCHN (*n* = 17)
**Median age, years (range)**	68 (38–89)	68 (38–89)	71 (39–87)
** >65, *n* (%)**	93 (57.4)	81 (56.3)	11 (64.7)
**Sex, *n* (%)**			
** Male**	114 (70.4)	107 (74.3)	7 (41.2)
** Female**	48 (29.6)	37 (25.7)	10 (58.8)
**ECOG-PS, *n* (%)**			
** 0**	78 (48.2)	69 (47.9)	9 (52.9)
** 1**	78 (48.2)	70 (48.6)	8 (47.1)
** 2**	5 (3.1)	5 (3.5)	0
** Unknown**	1 (0.6)	0	0
**Tumor location, *n* (%)**			
** Cervical lymph node**	52 (29.2)	49 (30.8)	3 (16.7)
** Hypopharynx**	24 (13.5)	24 (15.1)	0
** Oral (excluding tongue)**	20 (11.2)	19 (12.0)	1 (5.6)
** Oropharynx**	18 (10.1)	18 (11.3)	0
** Larynx**	11 (6.2)	11 (6.9)	0
** External auditory canal**	10 (5.6)	10 (6.3)	0
** Maxillary sinus**	6 (3.4)	6 (3.8)	0
** Nasopharynx**	5 (2.8)	2 (1.3)	3 (16.7)
** Maxilla**	5 (2.8)	4 (2.5)	1 (5.7)
** Parotid gland**	5 (2.8)	1 (0.6)	4 (22.2)
** Orbit**	4 (2.3)	3 (1.9)	1 (5.7)
** Parapharyngeal space**	3 (1.7)	2 (1.3)	1 (5.7)
** Tongue**	3 (1.7)	3 (1.9)	0
** Brain**	1 (0.6)	NA	NA
** Others**	11 * (6.2)	7 ** (4.4)	4 *** (22.2)
**TNM classification, *n* (%)**			
** T1–2**	48 (30.3)	43 (29.9)	5 (29.4)
** T3–4**	72 (45.6)	63 (43.8)	9 (52.9)
** N1–2**	33 (20.5)	31 (21.5)	2 (11.8)
** N3**	19 (11.8)	18 (12.5)	1 (5.9)
**Prior systemic therapy, *n* (%)**	128 (79.0)	120 (83.3)	8 (47.1)
**Prior radiation therapy, *n* (%)**	151 (93.2)	136 (94.4)	14 (82.4)
** Median cumulative dose,** ** Gy (range)**	70 (24–130)	70 (24–130)	68 (48–90)
**Duration from last irradiation**			
** <6 months, *n* (%)**	20 (13.3)	19 (14.0)	1 (7.1)
** ≥6 months, *n* (%)**	128 (84.8)	114 (83.8)	13 (92.9)
** Unknown**	3 (2.0)	3 (2.2)	0

* Included “neck (detail unspecified)” in two cases, and “nasal cavity”, “ethmoid sinus”, “pterygopalatine fossa”, “mandible”, “buccal area (detail unspecified)”, “eyelid”, “nasolacrimal canal”, “sublingual gland”, and “superior mediastinum” in one case each. ** Included “neck (detail unspecified)” in 2 cases, and “nasal cavity”, “pterygopalatine fossa”, “mandible”, “buccal area (detail unspecified)”, “superior mediastinum” in one case each. *** Included “ethmoid sinus”, “eyelid”, “nasolacrimal canal”, and “sublingual gland” in one case each. Abbreviations: SCCHN, squamous cell carcinoma of the head and neck; NSCCHN, non-squamous cell carcinoma of the head and neck; ECOG-PS, Eastern Cooperative Oncology Group Performance Status.

**Table 3:** Treatment parameters of BNCT for HNC patients.

Parameter	All (*N* = 160)	SCCHN (*n* = 143)	NSCCHN (*n* = 17)
**^10^B conc. after 2-h infusion, ppm (range)**	31.3 (20.9–46.5)	31.1 (21.0–46.5)	32.3 (20.9–40.7)
**Completion of planned irradiation, *n* (%)**			
** Completed**	155 (96.9)	138 (96.5)	17 (100)
** Interrupted**	5 (3.1)	5 (3.5)	0
**Actual charge amount of accelerator,** **C (interquartile range)**	2.44 (2.05–2.95)	2.45 (2.08–3.03)	2.29 (2.03–2.59)
**Prescribed dose of mucosa, *n* (%)**			
** <12 Gy-Eq**	14 (8.8)	11 (7.7)	3 (17.7)
** 12 Gy-Eq**	60 (37.5)	53 (37.1)	7 (41.2)
** >12 Gy-Eq**	86 (53.8)	79 (55.2)	7 (41.2)
**Tumor dose, Gy-Eq (interquartile range)**			
** D_max_**	46.9 (38.9–56.1)	46.4 (38.7–55.8)	50.3 (42.7–71.7)
** D_min_**	26.9 (22.4–32.2)	26.9 (22.2–31.7)	25.5 (24.1–37.8)
** D_mean_**	38.7 (32.3–45.3)	38.4 (32.4–44)	44.6 (31.2–57.4)

Abbreviations: BNCT, boron neutron capture therapy; HNC, head and neck cancer; SCCHN, squamous cell carcinoma of the head and neck; NSCCHN, non-squamous cell carcinoma of the head and neck; conc., concentration; D_max_, maximum dose; D_min_, minimum dose; D_mean_, mean dose.

**Table 4:** Acute TRAEs (with frequency ≥ 5% for Grade 1 and 2 toxicity).

Acute TRAE (*N* = 162)	Grade 1 and 2 *n* (%)	Grade 3 *n* (%)	Grade 4 *n* (%)
Hyperamylasemia *^1^	26 (16.1)	63 (38.9)	47 (29.0)
Stomatitis *^2^	66 (40.7)	17 (10.5)	0
Sialoadenitis *^3^	81 (50.0)	1 (0.6)	0
**Alopecia**	80 (49.4)	0	0
Decreased appetite *^4^	53 (32.7)	4 (2.5)	0
**Nausea**	48 (29.6)	2 (1.2)	0
**Taste disorder**	39 (24.1)	0	0
**Crystalluria**	33 (20.4)	0	0
Pharyngeal inflammation *^5^	28 (17.3)	2 (1.2)	0
Thirst *^6^	24 (15)	0	0
**Dysphagia**	15 (9.3)	8 (4.9)	0
**Malaise**	15 (9.3)	1 (0.6)	0
**Vomiting**	16 (9.9)	0	0
**Conjunctivitis**	14 (8.6)	0	0
Tumour pain *^7^	14 (8.6)	0	0
**Pyrexia**	13 (8.0)	0	0
Facial oedema *^8^	12 (7.4)	0	0
Dermatitis *^9^	7 (4.3)	1 (0.6)	0
**Pneumonia aspiration**	0	4 (2.5)	0
Renal impairment *^10^	1 (0.6)	2 (1.2)	0
Anaemia *^11^	1 (0.6)	2 (1.2)	0
**Dehydration**	0	2 (1.2)	0
**Diarrhoea**	1 (0.6)	1 (0.6)	0
Skin disorder *^12^	1 (0.6)	1 (0.6)	0
**Septic shock**	0	0	1 (0.6)
**Anaphylactic shock**	0	0	1 (0.6)
**Tracheal stenosis**	0	0	1 (0.6)
**Obstructive airways disorder**	0	0	1 (0.6)
**Cellulitis**	0	1 (0.6)	0
**Acinetobacter infection (site unknown)**	0	1 (0.6)	0
**Tumour necrosis**	0	1 (0.6)	0
**Malnutrition**	0	1 (0.6)	0
**Disorientation**	0	1 (0.6)	0
**Hypertension**	0	1 (0.6)	0
**Laryngeal necrosis**	0	1 (0.6)	0
**Mucosal ulceration**	0	1 (0.6)	0

*^1^ Including the description “amylase increased”. *^2^ Including the descriptions “gingival swelling” and “glossitis”. *^3^ Including the description “parotitis”. *^4^ Including the descriptions “hypophagia” and “eating disorder”. *^5^ Including the descriptions “pharyngeal erythema” and “pharyngeal erosion”. *^6^ Including the description “dry mouth”. *^7^ Including the description “cancer pain”. *^8^ Including the description “swelling face”. *^9^ Including the description “erythema”. *^10^ Including the descriptions “acute kidney injury”. *^11^ Including the descriptions “Iron deficiency anaemia” and “radiation anaemia”. *^12^ Including the description “skin ulcer”. All notations conform to MedDRA/J version 25.1. Abbreviations: TRAEs, treatment-related adverse events; MedDRA, Medical Dictionary for Regulatory Activities.

**Table 5:** Late TRAEs (with frequency ≥ 1% for Grade 1 + 2 toxicity).

Late TRAE (*N* = 157)	Grade 1 and 2 *n* (%)	Grade 3 *n* (%)	Grade 4 *n* (%)
**Dysphagia**	4 (2.6)	3 (1.9)	0
Thirst *^1^	4 (2.6)	0	0
Skin disorder *^2^	1 (0.6)	2 (1.3)	0
**Soft tissue infection**	0	2 (1.3)	0
**Hyperamylasemia**	0	2 (1.3)	0
**Papilloedema**	0	2 (1.3)	0
**Visual acuity reduced**	2 (1.3)	0	0
**Radiation necrosis (site unknown)**	2 (1.3)	0	0
Decreased appetite *^3^	2 (1.3)	0	0
**Pneumonia aspiration**	0	0	1 (0.6)
**Abscess bacterial (site unknown)**	0	1 (0.6)	0
**Brain abscess**	0	1 (0.6)	0
**Infected neoplasm**	0	1 (0.6)	0
**Cranial nerve paralysis**	0	1 (0.6)	0
**Cataract**	0	1 (0.6)	0
**Pneumonitis**	0	1 (0.6)	0
**Oroantral fistula**	0	1 (0.6)	0
**Ulcer haemorrhage**	0	1 (0.6)	0
**Radiation retinopathy**	0	1 (0.6)	0
**Osteoradionecrosis**	0	1 (0.6)	0

*^1^ Including the description “dry mouth”. *^2^ Including the descriptions “skin exfoliation” and “skin ulcer”. *^3^ Including the description “hypophagia”. All notations conform to MedDRA/J version 25.1. Abbreviations: TRAEs, treatment-related adverse events; MedDRA, Medical Dictionary for Regulatory Activities.

**Table 6:** Response rate in patients with SCCHN and NSCCHN.

	SCCHN (*n* = 137)	NSCCHN (*n* = 17)
**ORR, % (95% CI)**	72.3 (64.0–79.6)	64.7 (38.3–85.8)
**Best overall response**		
**CR, *n* (%)**	63 (46.0)	8 (47.1)
**PR, *n* (%)**	36 (26.3)	3 (17.7)
**SD, *n* (%)**	31 (22.6)	5 (29.4)
**PD, *n* (%)**	6 (4.4)	0
**NE, *n* (%)**	1 (0.7)	1 (5.9)

Tumor response in patients receiving post-treatment intervention was also included in the analysis. Abbreviations: SCCHN, squamous cell carcinoma of the head and neck; NSCCHN, non-squamous cell carcinoma of the head and neck; ORR, overall response rate; CI, confidence interval; CR, complete response; partial response; SD, stable disease; PD, progressive disease; NE, not evaluated.

**Table 7:** Comparison of tumor response between SCCHN with and without lymph node metastases.

	Without N (*n* = 91)	With N (*n* = 46)
**ORR, % (95% CI)**	75.8 (65.7–84.2)	65.2 (49.8–78.6)
**Best overall response**		
**CR, *n* (%)**	46 (50.6)	17 (36.7)
**PR, *n* (%)**	23 (25.3)	13 (28.3)
**SD, *n* (%)**	17 (18.7)	14 (30.4)
**PD, *n* (%)**	5 (5.2)	1 (2.2)
**NE, *n* (%)**	0	1 (2.2)

Tumor response in patients receiving post-treatment intervention was also included in the analysis. Abbreviations: SCCHN, squamous cell carcinoma of the head and neck; ORR, overall response rate; CI, confidence interval; CR, complete response; partial response; SD, stable disease; PD, progressive disease; NE, not evaluated.

## Data Availability

Data are contained within the article.
