# Correction: Sato et al. Safety of Boron Neutron Capture Therapy with Borofalan(^10^B) and Its Efficacy on Recurrent Head and Neck Cancer: Real-World Outcomes from Nationwide Post-Marketing Surveillance. *Cancers* 2024, *16*, 869

**DOI:** 10.3390/cancers16193297

**Published:** 2024-09-27

**Authors:** Mariko Sato, Katsumi Hirose, Satoshi Takeno, Teruhito Aihara, Keiji Nihei, Yoshihiro Takai, Toshimitsu Hayashi, Kosuke Bando, Hitomi Kimura, Keisuke Tsurumi, Koji Ono

**Affiliations:** 1 Department of Radiation Oncology, Hirosaki University Graduate School of Medicine, 5 Zaifu-cho, Hirosaki 036-8562, Japan; s_mariko@hirosaki-u.ac.jp; 2 Department of Radiation Oncology, Southern Tohoku BNCT Research Center, 7-10 Yatsuyamada, Koriyama 963-8052, Japan; y-takai@hirosaki-u.ac.jp; 3 Kansai BNCT Medical Center, Osaka Medical and Pharmaceutical University, 2-7 Daigaku-machi, Takatsuki 569-8686, Japan; satoshi.takeno@ompu.ac.jp (S.T.); teruhito.aihara@ompu.ac.jp (T.A.); keiji.nihei@ompu.ac.jp (K.N.); 4 Department of Radiation Oncology, Osaka Medical and Pharmaceutical University, 2-7 Daigaku-machi, Takatsuki 569-8686, Japan; 5 Department of Otolaryngology Head and Neck Surgery, Osaka Medical and Pharmaceutical University, 2-7 Daigaku-machi, Takatsuki 569-8686, Japan; 6 Stella Pharma Corporation, ORIX Kouraibashi Building, 3-2-7 Kouraibashi, Chuo-ku, Osaka 541-0043, Japan; hayashi@stella-pharma.co.jp (T.H.); bando@stella-pharma.co.jp (K.B.); kimura@stella-pharma.co.jp (H.K.); 7 Sumitomo Heavy Industries, Ltd., 5-2 Soubirakichou, Niihama 792-0001, Japan; keisuke.tsurumi@shi-g.com; 8 BNCT Joint Clinical Institute, Osaka Medical and Pharmaceutical University, 2-7 Daigaku-machi, Takatsuki 569-8686, Japan; koji.ono@ompu.ac.jp

The authors wish to make the following corrections to this paper [1].

The tumor responses using RECIST to calculate the response rates reported in this paper were found to be based on data compiled with and without post-treatment intervention. A sentence in “2.4. Assessments” and legends in Tables 6 and 7 were corrected to clarify that the response rates in this paper are based on data that include the impact of post-treatment interventions. We recognize that this correction will result in a significant change in the interpretation of the response rate. However, on the other hand, there are no corrections to the survival data presented in this article, and we believe that the significance of this article will be preserved.

The following correction was made to “2.4. Assessments”, paragraph 2:

Secondary efficacy endpoints included tumor response and overall survival (OS). Tumor response was based on RECIST version 1.1 using consistent imaging with CT or MRI. The best overall response of lesions treated with BNCT was recorded at any point within six months after BNCT. The objective response rate (ORR) was evaluated as the proportion of patients showing CR or partial response (PR). Tumor response in patients receiving post-treatment intervention was also included in the analysis.

The correct legends in Tables 6 and 7 appear below.

**Correct legend to Table 6**

“Tumor response in patients receiving post-treatment intervention was also included in the analysis. Abbreviations: SCCHN, squamous cell carcinoma of the head and neck; NSCCHN, non-squamous cell carcinoma of the head and neck; ORR, overall response rate; CI, confidence interval; CR, complete response; partial response; SD, stable disease; PD, progressive disease; NE, not evaluated.”

**Correct legend to Table 7**

“Tumor response in patients receiving post-treatment intervention was also included in the analysis. Abbreviations: SCCHN, squamous cell carcinoma of the head and neck; ORR, overall response rate; CI, confidence interval; CR, complete response; partial response; SD, stable disease; PD, progressive disease; NE, not evaluated.”

In regard to the above corrections, the Abstract was also corrected:

**Abstract**: Background: This study was conducted to evaluate the real-world safety and efficacy of boron neutron capture therapy (BNCT) with borofalan(^10^B) in Japanese patients with locally advanced or locally recurrent head and neck cancer (LA/LR-HNC). Methods: This prospective, multicenter observational study was initiated in Japan in May 2020 and enrolled all patients who received borofalan(^10^B) as directed by regulatory authorities. Patient enrollment continued until at least 150 patients were enrolled, and adverse events attributable to drugs, treatment devices, and BNCT were evaluated. The patients with LA/LR-HNC were systematically evaluated to determine efficacy. Results: The 162 patients enrolled included 144 patients with squamous cell carcinoma of the head and neck (SCCHN), 17 patients with non-SCCHN (NSCCHN), and 1 patient with glioblastoma. Treatment-related adverse events (TRAEs) were hyperamylasemia (84.0%), stomatitis (51.2%), sialoadenitis (50.6%), and alopecia (49.4%) as acute TRAEs and dysphagia (4.5%), thirst (2.6%), and skin disorder (1.9%) as more common late TRAEs. One- and two-year OS rates in patients with recurrent SCCHN were 78.8% and 60.7%, respectively. Conclusions: This post-marketing surveillance confirmed the safety and efficacy of BNCT with borofalan(^10^B) in patients with LA/LR-HNC in a real-world setting.

In addition, the baseline characteristics of patients for analysis are shown in Table 2. Although values regarding the duration from the last irradiation were recorded in units of years, they were analyzed under the misapprehension that they were presented in units of months, and the incorrect results were published. Additionally, there was a numerical error in the count regarding the age of the subject with squamous cell carcinoma of the head and neck.

The corrected baseline characteristics (Table 2) appear below.

**Table 2:** Baseline characteristics.

Characteristic	All (*n* = 162)	SCCHN (*n* = 144)	NSCCHN (*n* = 17)
**Median age, years (range)**	68 (38–89)	68 (38–89)	71 (39–87)
**>65, *n* (%)**	93 (57.4)	81 (56.3)	11 (64.7)
**Sex, *n* (%)**			
**Male**	114 (70.4)	107 (74.3)	7 (41.2)
**Female**	48 (29.6)	37 (25.7)	10 (58.8)
**ECOG-PS, *n* (%)**			
**0**	78 (48.2)	69 (47.9)	9 (52.9)
**1**	78 (48.2)	70 (48.6)	8 (47.1)
**2**	5 (3.1)	5 (3.5)	0
**Unknown**	1 (0.6)	0	0
**Tumor location, *n* (%)**			
**Cervical lymph node**	52 (29.2)	49 (30.8)	3 (16.7)
**Hypopharynx**	24 (13.5)	24 (15.1)	0
**Oral (excluding tongue)**	20 (11.2)	19 (12.0)	1 (5.6)
**Oropharynx**	18 (10.1)	18 (11.3)	0
**Larynx**	11 (6.2)	11 (6.9)	0
**External auditory canal**	10 (5.6)	10 (6.3)	0
**Maxillary sinus**	6 (3.4)	6 (3.8)	0
**Nasopharynx**	5 (2.8)	2 (1.3)	3 (16.7)
**Maxilla**	5 (2.8)	4 (2.5)	1 (5.7)
**Parotid gland**	5 (2.8)	1 (0.6)	4 (22.2)
**Orbit**	4 (2.3)	3 (1.9)	1 (5.7)
**Parapharyngeal space**	3 (1.7)	2 (1.3)	1 (5.7)
**Tongue**	3 (1.7)	3 (1.9)	0
**Brain**	1 (0.6)	NA	NA
**Others**	11 * (6.2)	7 ** (4.4)	4 *** (22.2)
**TNM classification, *n* (%)**			
**T1–2**	48 (30.3)	43 (29.9)	5 (29.4)
**T3–4**	72 (45.6)	63 (43.8)	9 (52.9)
**N1–2**	33 (20.5)	31 (21.5)	2 (11.8)
**N3**	19 (11.8)	18 (12.5)	1 (5.9)
**Prior systemic therapy, *n* (%)**	128 (79.0)	120 (83.3)	8 (47.1)
**Prior radiation therapy, *n* (%)**	151 (93.2)	136 (94.4)	14 (82.4)
** Median cumulative dose,** ** Gy (range)**	70 (24–130)	70 (24–130)	68 (48–90)
**Duration from last irradiation**			
**<6 months, *n* (%)**	20 (13.3)	19 (14.0)	1 (7.1)
**≥6 months, *n* (%)**	128 (84.8)	114 (83.8)	13 (92.9)
**Unknown**	3 (2.0)	3 (2.2)	0

* Included “neck (detail unspecified)” in two cases, and “nasal cavity”, “ethmoid sinus”, “pterygopalatine fossa”, “mandible”, “buccal area (detail unspecified)”, “eyelid”, “nasolacrimal canal”, “sublingual gland”, and “superior mediastinum” in one case each. ** Included “neck (detail unspecified)” in 2 cases, and “nasal cavity”, “pterygopalatine fossa”, “mandible”, “buccal area (detail unspecified)”, “superior mediastinum” in one case each. *** Included “ethmoid sinus”, “eyelid”, “nasolacrimal canal”, and “sublingual gland” in one case each. Abbreviations: SCCHN, squamous cell carcinoma of the head and neck; NSCCHN, non-squamous cell carcinoma of the head and neck; ECOG-PS, Eastern Cooperative Oncology Group Performance Status.

The authors would like to apologize for any inconvenience caused to the readers by these changes and state that the scientific conclusions are unaffected. This correction was approved by the Academic Editor. The original article has been updated.
